# Large-scale STI services in Avahan improve utilization and treatment seeking behaviour amongst high-risk groups in India: an analysis of clinical records from six states

**DOI:** 10.1186/1471-2458-11-S6-S10

**Published:** 2011-12-29

**Authors:** Anup Gurung, Prakash Narayanan, Parimi Prabhakar, Anjana Das, Virupax Ranebennur, Saroj Tucker, Laxmi Narayana, Radha R, K Prakash, Touthang, Collins Z Sono, Teodora Wi, Guy Morineau, Graham Neilsen

**Affiliations:** 1 FHI, India Country Office, India; 2 India HIV/AIDS Alliance, India; 3 Hindustan Latex Family Planning Promotion Trust, India; 4 Tamil Nadu AIDS Initiative, India; 5 Karnataka Health Promotion Trust, India; 6 Emmanuel Hospital Association, India; 7 World Health Organization, Regional Office for the Western Pacific (Formerly with FHI, India Country Office; 8 FHI, Asia Pacific Regional Office, Thailand; 9 Independent Consultant (Formerly with FHI, Asia Pacific Regional Office, Thailand

## Abstract

**Background:**

Avahan, the India AIDS Initiative, implemented a large HIV prevention programme across six high HIV prevalence states amongst high risk groups consisting of female sex workers, high risk men who have sex with men, transgenders and injecting drug users in India. Utilization of the clinical services, health seeking behaviour and trends in syndromic diagnosis of sexually transmitted infections amongst these populations were measured using the individual tracking data.

**Methods:**

The Avahan clinical monitoring system included individual tracking data pertaining to clinical services amongst high risk groups. All clinic visits were recorded in the routine clinical monitoring system using unique identification numbers at the NGO-level. Visits by individual clinic attendees were tracked from January 2005 to December 2009. An analysis examining the limited variables over time, stratified by risk group, was performed.

**Results:**

A total of 431,434 individuals including 331,533 female sex workers, 10,280 injecting drug users, 82,293 men who have sex with men, and 7,328 transgenders visited the clinics with a total of 2,700,192 visits. Individuals made an average of 6.2 visits to the clinics during the study period. The number of visits per person increased annually from 1.2 in 2005 to 8.3 in 2009. The proportion of attendees visiting clinics more than four times a year increased from 4% in 2005 to 26% in 2009 (p<0.001). The proportion of STI syndromes diagnosed amongst female sex workers decreased from 39% in 2005 to 11% in 2009 (p<0.001) while the proportion of STI syndromes diagnosed amongst high risk men who have sex with men decreased from 12% to 3 % (p<0.001). The proportion of attendees seeking regular STI check-ups increased from 12% to 48% (p<0.001). The proportion of high risk groups accessing clinics within two days of onset of STI-related symptoms and acceptability of speculum and proctoscope examination increased significantly during the programme implementation period.

**Conclusions:**

The programme demonstrated that acceptable and accessible services with marginalised and often difficult–to-reach populations can be brought to a very large scale using standardized approaches. Utilization of these services can dramatically improve health seeking behaviour and reduce STI prevalence.

## Background

In India, high risk groups (HRG) which include female sex workers (FSWs), men who have sex with men (MSM), injecting drug users (IDUs) and transgenders (TGs) have high HIV prevalence based on data from the national sentinel surveillance 2009 and other studies [1-3]. Concentrated epidemics amongst HRGs require an integrated service approach for HIV prevention efforts [4-6]. Avahan is a large scale HIV prevention programme in six states of India which have a population of about 300 million. The main components of the Avahan intervention for HRGs are peer-led outreach education, condom promotion and distribution, clinical services for managing sexually transmitted infections (STIs), community empowerment and structural interventions.

High burden of STIs amongst HRGs has been reported from most developing nations [7]. Improved primary care of symptomatic and asymptomatic STIs through programmes targeting vulnerable groups has been promoted globally to achieve scaling-up, high coverage and decrease in STI burden [8-10]. STI prevention amongst high risk groups is the cornerstone for prevention efforts in concentrated HIV epidemics [11,12].

Provision of quality STI services to HRGs is low in developing countries [13]. Developing acceptable, accessible STI services for HRGs in resource-constrained settings has been challenging due to prevalent stigma, discrimination, socio-cultural barriers and affordability issues. Studies have demonstrated that when STI services are designed to address these issues, utilization by HRGs improves [14]. To further improve service utilization and health seeking behaviour, standardized treatment guidelines, a robust STI service delivery system, regular supervision and tracking coverage are critical. However, until now interventions tracking coverage using unique identifiers or individual tracking systems amongst HRGs have been reported rarely and at small scale [15].

We report here an analysis using routine clinical monitoring data which is based on individual tracking of HRGs in Avahan. The analysis focuses on STI services provided to HRGs; assessing utilization, health seeking behaviour and STI syndrome trends between 2005 and 2009.

## Methods

### The programme

Avahan is an HIV prevention programme for FSWs, MSM, TGs and IDUs implemented in six states of India with historically the highest prevalence of HIV: Andhra Pradesh, Tamil Nadu, Maharashtra, Karnataka, Manipur and Nagaland [16]. The programme is implemented by seven lead implementing partners through a network of about 130 local non-governmental (NGOs) and community-based organizations (CBOs) from 2004 to date. While the drivers of the epidemic in the four southern states are mainly sexual, the epidemics in the two north-eastern states are driven by injecting drug use [10].

At the individual level, primary and secondary prevention of STIs is a key Avahan strategy. Avahan-supported clinics provide STI services to HRGs in the six intervention states .In addition, the clinical services in the two north eastern states also address injection related infections with less emphasis on STIs compared to the southern states. Avahan-supported STI services are standardized across the states in which an essential STI service package was defined [17]. The package consisted of syndromic management of symptomatic infections as per Indian national guidelines; presumptive treatment for gonorrhoea and chlamydia at the first visit which was repeated if the individual had not attended the clinic for any STI check ups in the previous six months; quarterly STI check ups and six-monthly syphilis screening. IDUs were provided services for symptomatic STIs and injected related infections.

### Routine clinical monitoring data base

A component of the monitoring system was individual tracking data pertaining to registration and clinical services. At the NGO-level (NGOs provided service coverage at the district level), each individual was provided a unique identification number which was based on the project identification number, the district code and the number of the peer educator responsible for that individual. This unique identifier was used to maintain paper-based NGO clinic records for the individual over time. Information for each clinical visit was recorded in a standardized clinical encounter form, entered into a computerized database by the NGOs/CBOs and collated electronically by the lead implementing partners. Quality control of the database was maintained by the individual implementing units and state lead partners.

### Individual STI tracking data

Individual STI data was collated from all lead implementing partners to create the pan-Avahan individual tracking STI database. Data variables from the database and their description are listed in Table 1.

**Table 1:** Variables used from the individual tracking data

Area	Variable name	Definition	Remarks
Unique identifiers	IP name	Name of the lead implementing partner	
	Project name	Name of implementing NGO	830 records without project name were deleted during cleaning
	Registration date	Date of registration of HRG individual with NGO	4,500 records without registration date were removed from the master database
	ID number	Unique tracking ID entered in the registration file	55,000 records without ID number were removed from the master database during cleaning
	Target group	Target group defined by the typology of HRG, FSW, MSM, TG, IDU, clients and regular partners	200 records without target groups were grouped as others
	Sub target group (place of solicitation for FSW, self-identity for MSM)	Defined by the sub-population within the target groups of HRG: FSW – home-based, street-based, bar-based, brothel-based, lodge-based, highway-based. MSM - kothi, panthi, double decker, Transgender, clients/regular partners, IDUs	Re-grouped the local terminology to make this uniform
	Visit date	Date of current visit to the clinic	2200 records without visit dates were removed during cleaning of the data base

Demographics	Sex	Gender of the STI patient: male, female or transgender	
	Age	Age in completed years on the first clinic visit	
	Number of years in sex work	Number of years into sex work (recorded at the time of registration)	
	Number of clients	Number of clients in last week (recorded at the time of first visit)	

Clinic visits	Symptoms visit	“STI symptoms visit” - reason for visit to the clinic was STI related symptoms	
	Regular STI checkup	“STI check-up” - the individual does not complain of STI symptoms but receives genital examination which may include speculum or proctoscope examination and/or STI Treatment	
	Follow-up visit	“STI follow-up” - the individual returned to the clinic within two weeks of last treatment for a review by the doctor	
	General visit	General health visit – visit for services other than STI related	
	First STI clinic visit	First STI visit to the clinic ever	
	Duration of symptoms (days)	Period the patient is suffering from current/longest running STI symptom	Information in actual days as well as in codes - was changed to codes
	Internal examination	Whether speculum or proctoscopic examination conducted during STI consultation	

Syndrome diagnosed	Vaginal cervical discharge	Female with vaginal or cervical discharge on examination	
	Genital ulcer disease	Female or male with genital or ano-rectal ulceration with or without blisters	
	Lower abdominal pain	Female has lower abdominal pain or tenderness, or cervical motion tenderness	
	Urethral discharge	Male with urethral discharge with or without dysuria	
	Ano-rectal discharge	Male with symptoms of tenesmus or if ano-rectal discharge seen on exam	Females with ano-rectal discharge were also recorded as such.
	Other syndromes	Other STI syndromes (e.g. inguinal bubo, genital warts, scrotal swelling, genital scabies etc.)	
	Asymptomatic	Asymptomatic treatment is given	
	None	Asymptomatic treatment is given	

Treatment packs	Coded in packs**	Specific drugs for specific syndromes as per clinical operating guidelines	

Locally described typologies from across the states were merged to define uniform typologies across Avahan. The typologies mainly reflected the place of solicitation for FSWs and were classified as such. Street based FSWs were those who solicited clients on the streets; while home based FSWs solicited clients at home, similarly bar based FSWs were found mainly soliciting in bars; and brothel based FSWs soliciting points were organized brothels. Amongst MSM, the typologies were classified as per self-reported sexual identity generally based on roles in anal sex. “*Kothis*” were self-identified anal receptive MSM, “*Panthis*” were self-identified anal insertive partners and “*Double-deckers*” were self identified anal insertive as well as anal receptive partners.

### Data sources include

(1) Avahan programme generated HRG size estimates: at the start of the programme in a district or sub-district, NGOs conducted a formal external mapping and size estimation exercise. Some state-level lead implementing partners updated these numbers on a regular basis (every 12 to 18 months) using programme data; others conducted periodic formal size estimation exercises. Size estimates were done separately for FSWs, MSM, IDUs and TGs and were available consistently from 2007 onwards [18,19].

(2) Data from clinical encounter forms at the level of individual clinics: registration details obtained at the first clinic visit included including age, sex and typology. At this time a unique clinic number was assigned. Clinical encounter forms labelled with the individual’s clinic number were filled for each clinic visit.

The merged database of registrations and subsequent clinical visits were cleaned in consultation with the lead implementing agencies. During the cleaning process clinical encounter forms missing unique clinic numbers or dates of visits were deleted (for details, see Table 1). In the case of the variable, duration of symptoms prior to clinic visit, the format for recording was changed from a categorical to a continuous variable in 2007. Data was recoded to categorical variables to utilize all years of data.

### Analytical approach

The data was cleaned and merged using MS-Access 2003™ and analyzed using statistical package STATA™ version 10. Four broad areas of enquiry formed the basis of the analysis of the paper. The sections below describe in details the analytical approaches and data sources:

#### (1) Demography and proportion of HRGs reaching the clinics

Age at first clinic visit, number of years into sex work, number of clients per week by typology were analyzed. The proportion of HRGs using clinical services was estimated using the number of individuals from clinic records and the estimated denominator from the programme. This analysis for the clinical coverage of HRGs was restricted to the period 2007 to 2009 as the previous years were still being utilized to increase the scale of services.

#### (2) Utilization of the clinics

The analysis included the number of visits per year by individuals. Trends in number of repeat visits by HRGs by typology over the programme implementation period were analyzed, adjusted for age. “STI visits”, were defined as visits by HRGs who attended the clinics for relevant symptoms, regular-check ups or follow up visits within 14 days of a previous symptomatic visit.

#### (3) Improving health seeking behaviour and changing trends in STI syndromes

Treatment seeking behaviour was analyzed by duration of symptoms prior to reporting to the clinics, trends in regular STI check ups and internal examination. STI syndromes diagnosed and trends were analyzed by adjusting confounders which included age and sub-population groups. “Cohorts for the year”, were defined as those HRGs coming to clinics for the first time in that year and followed up subsequently.

#### (4) Quality of clinical services provided

Prescription analysis of correct treatment given as per standard packs described in the Avahan Clinic Operating Guidelines and Standards (COGS) were analyzed.

## Results

A total of 431,434 high risk individuals made 2.7 million visits to the targeted programme clinics in the six states. The HRGs consisted of 331,533 FSWs, 10,280 IDUs, 82,293 MSM and 7,328 TGs.

The age distribution of HRGs varied by typology at their first clinic visit, as shown in Table 2.Amongst FSWs, the bar-based were the youngest with a mean age of 24.7 years (SD 5.0); among the MSM the youngest were “*Panthis*” with a mean age of 27.8 years (SD 7.7).The reported number of commercial clients in the previous week was significantly higher amongst the highway-based FSWs who had an average of 12 clients per week; followed by the brothel-based (eight clients per week) and street-based (six clients per week). Amongst the MSM who reported selling sex, “*double-deckers”* had the highest number of partners with four clients per week. The mean number of years in sex work for FSWs and MSM were 3.1 (SD 4.4) and 1.8 (SD 3.6) respectively.

**Table 2:** Age and sex work characteristics by high risk group and typology of clinic attendees

High risk group typology**	Number of individual HRG	Mean age in years (SD*)	Reported clients per week (SD)	Reported number years in sex work (SD)
FSW	331,533	29.8 (6.6)	4.4 (8.0)	3.1 (4.4)

Bar-based	35,584	24.7 (5.0)	1.3 (2.8)	1.9 (3.4)
Brothel-based	23,864	28.8 (6.5)	7.6 (14)	2.0 (4.5)
Highway-based	2,253	28.6 (6.0)	11.8 (18)	2.6 (3.3)
Home-based	116,516	30.3 (6.4)	3.9 (6.5)	3.5 (4.9)
Lodge-based	9,514	29.4 (7.0)	3.5 (7.2)	2.7 (3.6)
Street-based	112,384	30.8 (6.3)	6.1 (8.8)	3.7 (4.1)

MSM	82,293	28.8 (8.0)	3.4 (12.4)	1.8 (3.6)

*Double decker*	22,390	29.4 (7.7)	4.3 (13)	2.3 (4.1)
*Kothi*	35,961	29.4 (8.2)	3.9 (14)	1.8 (3.9)
*Panthi*	12,025	27.8 (7.7)	0.1 (3.6)	0.03 (0.4)

TG	7,328	29.9 (8.9)	2.8 (8.9)	1.6 (3.7)

IDU	10,280	27.1(5.6)	Not applicable	Not applicable

* SD - standard deviation.

** Typology - for FSW is defined by the place of solicitation for sex work: bar, brothel, highways, home, lodge or street-based. Typology for MSM is based on reported self identity of being receptive (Kothi), insertive (Panthi) or both (Double decker) during anal sex.

Individuals made an average of 6.2 visits per year to the clinics throughout the period. The average annual number of visits increased consistently: 1.7 visits in 2005 per individual; 3.1 visits in 2006; 5.7 visits in 2007; 9.1 visits in 2008; and 8.3 visits in 2009. The utilization by typology of HRG was not uniform with utilization percentages exceeding the estimated denominator in some populations and low utilization in others as shown in Table 3.

**Table 3:** Percentage of high risk groups who accessed clinical services

Typology of HRG	Percentage individual HRG who accessed clinics out of the estimated figures*			
Year	2007	2008	2009	p-values**

FSW (all)	68	78	100	<0.001

Brothel based	108	172	230	<0.001
Lodge based	90	94	181	<0.001
Bar based	82	101	161	<0.001
Street based	69	69	90	<0.001
Home based	75	85	97	<0.001

MSM (all)	43	54	79	<0.001

Kothi	40	48	68	<0.001
Panthi	98	108	121	<0.001
Double decker	57	73	113	<0.001

IDU***	14	21	24	<0.01

* Denominator used were the estimated figures of HRG calculated by the lead implementing partners for the year 2007,2008 & 2009 by typology.

** p values using X2.

*** IDU were asked to report to the clinics only if they had STI symptoms/general health ailments or IDU related problems like abscess unlike the other HRG who were mobilized to come for regular STI check ups.

*** Mapping data not available for year 2005 & 2006.

New sex workers (defined as new into the project as shown in Table 4) who accessed the Avahan clinics increased from 43, 394 in 2005 to 220,877 in 2008. However, retention within the cohorts by years of follow up showed a declining trend and ranged from 22% to 25% over the five year period of follow up.

**Table 4:** Dynamic cohort of high risk groups followed over the years in Avahan clinics

Follow up of annual cohorts**		% retained within the cohort by years of follow up^#^				
All HRG individuals	New Individual HRGs^&^ coming into the cohort	Year-zero	2nd year	3rd year	4th year	5th year
2005	43,394	100	51	39.7	31.6	24.2
2006	108,836	100	61.7	46.2	33.5	
2007	169.612	100	68.6	47.4		
2008	220,877	100	62			

MSM		Year-zero	2nd year	3rd year	4th year	5th year

2005	4,887	100	52	41.2	32.8	25.2
2006	15,833	100	63	46.7	33.5	
2007	29,848	100	68.9	47.1		
2008	42,084	100	63.5			

FSW		Year-zero	2nd year	3rd year	4th year	5th year

2005	36,757	100	46	36	28	22
2006	87,480	100	63	52	41	
2007	132,144	100	75	57		
2008	165,832	100	69			

Annual cohorts were defined as the new HRGs coming in the clinics in the particular year.

# % retained in the cohort is the persons retained in the follow period who belong to the annual cohort.

& new HRGs implies new to the clinics.

The number of repeat visits was found to increase consistently amongst FSWs, MSM and IDUs from 2005 to 2009 as shown in Table 5. HRGs who visited the clinics more than four times per year increased over the years. An increasing proportion of HRGs attended the clinics for regular STI check-ups during the period. The proportion of clinic attendees undergoing internal examination (i.e. vaginal speculum or proctoscopy) increased from 10 % to 53 % amongst FSWs, from 1% to 54% amongst MSM and from 0.7% to 27 % amongst TGs from the year 2005 to 2009. Treatment seeking behaviour improved with an increasing proportion of HRGs coming to the clinics within two days of the onset of symptoms.

**Table 5:** Increasing health seeking by category of HRG 2005-2009 adjusted for age and typology

Category/variable	2005	2006	2007	2008	2009	p-value*
Internal examination	% examined out of total visits					

FSW	10	28	42	48	53	<0.001
MSM	1	6	17	45	54	<0.001
TG	0.7	35	44	32	27	<0.001

Regular STI check ups	% reported for STI check ups out of total visits					

All HRG	12	31	45	50	48	<0.001
MSM	22	46	57	59	53	<0.001
FSW	10	28	42	48	47	<0.001
TG	29	56	61	72	71	<0.001

Treatment seeking behaviour	% reported within 2 days of onset of symptoms					

FSW with VCD^#^	7	13	20	22	32	<0.001
MSM with UD^§^	6	8	11	27	24	<0.001
IDU with UD	28	16	23	16	21	<0.001
TG with ARD^€^	21	11	43	56	35	<0.001

Treatment seeking behaviour	% reported within 3-7 days of onset of symptoms					

FSW with VCD	11	17	25	23	16	<0.001
MSM with UD	26	35	35	32	30	<0.001
IDU with UD	25	19	24	33	38	<0.001
TG with ARD	43	45	32	29	27	<0.001

Number of visits per year	% reported per year					

FSW N=331,616 individuals made 2,11,727 clinic visits	2005	2006	2007	2008	2009	p-value *

1 to 2 visits per year	79	50	31	20	21	<0.001
2 to 4 visits per year	14	22	21	18	13	<0.001
More than 4 visits per year	8	28	48	62	66	<0.001

HR-MSM N=82,246 made 5,25,862 clinic visits	% reported per year					

1 to 2 visits per year	72	53	35	22	22	<0.001
2 to 4 visits per year	14	20	21	19	13	<0.001
More than 4 visits per year	14	28	44	59	65	<0.001

TG N=7,330 individuals made 38,613 clinic visits	% reported per year					

1 to 2 visits per year	63	53	29	21	29	<0.001
2 to 4 visits per year	16	17	17	17	17	<0.001
More than 4 visits per year	22	30	54	62	54	<0.001

IDU N=10,280 individuals made 23990 clinic visits	% reported per year					

1 to 2 visits per year	96	91	75	44	64	<0.001
2 to 4 visits per year	4	8	17	20	14	<0.001
More than 4 visits per year	0.1	1	8	37	22	<0.001

* p values matched for age & typology.

# VCD Vaginal cervical discharge.

§ UD Urethral discharge.

€ ARD Ano rectal discharge.

**Figure 1:**
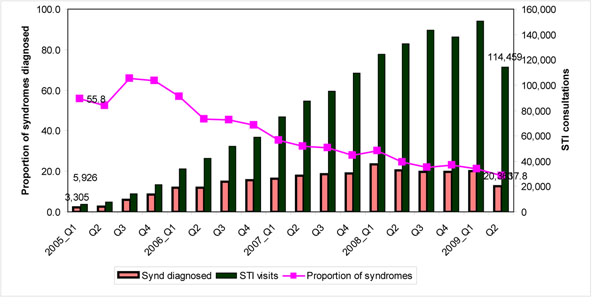
Decreasing trends of STI syndromes and improving health seeking behaviour Jan 2005 to June 2009.

There was a declining trend in the proportion of all syndromes diagnosed amongst HRGs from 2005 to 2009, as shown in Table 6. There was a decline in STI syndromes occurred amongst FSWs, MSM, TGs and IDU (p=<0.001). Amongst MSM self identity of being anal receptors, penetrates or both did not match the STI syndromes diagnosed. While the STI regular check up visits increased over the quarters of follow-up, there was a distinct decreasing trend in syndromes diagnosed, as shown in Fig -1.

**Table 6:** Trends of STI syndromes diagnosed amongst clinic attendees: 2005-2009, adjusted for age and typology

Category	2005	2006	2007	2008	2009	Odds ratio*
						
No of syndromes diagnosed						
	Percentage diagnosed out of total visits					

FSW						

VD (287,070)	30	24	16	12	10	0.7
GUD (11,913)	2	1	0.6	0.5	0.3	0.67
LAP (63,499)	10	6	4	3	2	0.63
Any STI^§^ (353,699)	39	30	20	15	11	0.67

MSM						

UD (16,574)	6.6	6.2	4.7	3.3	1.7	0.67
ARD (8,638)	2	3.2	1.7	1.9	1.2	0.79
GUD (5,191)	3.4	2.2	1.3	1.1	0.5	0.63
Any STI^&^ (30,242)	12	11	8	6	3	0.61

MSM Kothi**						

UD (6,964)	4.02	4.75	3.74	3.17	1.49	0.71
ARD (5471)	2.28	3.94	2.23	2.61	1.4	0.79

MSM Panthi^&&^						

UD (3,981)	25.55	15.68	8.55	3.95	2.2	0.48
ARD (764)	3.8	3.61	1.05	0.71	0.7	0.61
MSM Double decker^##^						
UD (4,201)	7.77	5.46	4.93	3.36	1.63	0.64
ARD (1,967)	1.58	2.62	1.39	1.8	1.01	0.81

TG						

Any STI (1,039)	0.2	0.2	0.2	0.1	0	0.92

IDU						

Any STI (6,267)	0.5	0.4	0.4	0.2	0.1	0.47

* p-value for all syndromes = <0.001.

^§^ Any STI FSW: includes vaginal discharge (VD), genital ulcer disease (GUD), lower abdominal pain (LAP), ano-rectal discharge, genital warts, genital scabies etc.

^&^ Any STI MSM/TG/IDU: includes urethral discharge (UD), ano-rectal discharge (ARD), genital ulcer disease (GUD), scrotal swelling, inguinal bubo, genital warts, genital scabies etc.

^**^ Kothi MSM who are self identified as anal receptive.

^&&^ Panthi MSM who self identified as anal penetrative.

^##^ Double decker MSM who self identify as both anal recpetors as well as penetrator.

Based on comparisons of the records of syndrome treatment packets prescribed and the recorded diagnosis, treatment was dispensed correctly 61% to 92% of times. On an average, correct treatment for FSWs with a diagnosis of lower abdominal pain was the poorest at 61%; FSWs with vaginal discharge were treated correctly over 90% of the time, as shown in Table 7.

**Table 7:** Correct treatment* for STI syndromes 2005-2009

	% who received correct treatment based on dispensing the correct syndrome packet					
HRG/ STI syndrome diagnosed**	2005	2006	2007	2008	2009	(mean of 2005 - 2009)

Clients or regular partners with urethral discharge^§^ (1339)	97.5	91.7	94.4	89.5	92.3	91.9
IDU with urethral discharge (41,709)	94.3	85.0	86.3	89.3	89.8	88.0
MSM with urethral discharge (16,574)	78.4	88.4	87.9	62.6	79.6	77.6
MSM with ano-rectal discharge^€^ (8,638)	57.9	86.6	86.0	49.2	90.6	74.1
TG with urethral discharge (362)	87.5	94.3	75.9	50.4	78.6	71.5
FSW with vaginal cervical discharge^#^ (287,070)	92.3	93.6	94.8	95.9	96.4	94.6
FSW with lower abdominal pain^¥^ (63,499)	57.3	63.0	62.3	56.4	67.6	61.3

* Correct treatment was defined as the correct treatment packets given as per the syndromes diagnosed during each clinic visit.

** STI syndrome diagnosed: all STI syndromes were diagnosed as per clinical guidelines.

§ Urethral discharge: defined as males with urethral discharge on clinical examination with or without dysuria.

€ Ano-rectal discharge: defined as symptoms of tenesmus and ano-rectal discharge seen on clinical examination.

# Vaginal cervical discharge: defined as female with vaginal or cervical discharge on examination.

¥ Lower abdominal pain: defined as female who has lower abdominal pain with or without cervical motion tenderness.

## Discussion

The analysis of five years of individual clinic tracking records of HRGs shows improved health seeking behaviour, declining trends in STI syndromes and increasing utilization of services provided by Avahan clinics across six states. More detailed analysis of the Avahan programme data and quality issues have been described in previous reports that showed a high level of infrastructure by Avahan and quality STI services [20]. These data indicate that quality STI services were brought to scale across the Avahan programme districts, resulting in reduced prevalence of STI syndromes amongst individuals attending the Avahan clinics.

Prevention of STI transmission to and from sex workers is critical to limiting the establishment and expansion of these epidemics at the population level. The role of HRGs and client groups in the epidemiology of a particular STI depends upon the frequency and nature of commercial sex transactions and the transmission dynamics of each STI. As STI treatment and prevention programmes improve in quality and expand in scope, the duration of infectiousness and perhaps the transmission efficacy of the targeted STI should decrease [21-23].

However, HRGs experience a high degree of social marginalization and discrimination in society especially from healthcare providers and therefore do not have adequate or equitable access to health services. Access to services can be a key motivator for many FSWs to interact with programme staff and to participate in programme activities [24].

Access to health services is determined by three factors, the health seeking behaviour of the population, the health care provider’s attitudes and the healthcare delivery systems [25,26]. In the present analysis, the healthcare seeking behaviour was studied amongst marginalized groups who showed improving health seeking behaviour reflected by the increased number of clinic visits, the increasing proportions coming for STI check-ups and early treatment seeking behaviour trends over the programme implementation period. Trends in utilization, however, did vary as per the typology of FSWs, MSM and IDUs. Recent research reveals a more complex picture of STI epidemiology amongst HRG [27]. This complexity is based on HRG populations who actually are diverse sub-populations within themselves, each with distinct population characteristics. In the present analysis brothel based female sex workers were easily accessible compared to the street based or home based sex workers. Amongst MSM the “*Kothis”* were less accessible compared to other typologies. Health seeking behaviour thus may be related to the population characteristics within sex worker groups.

Increasing proportions of internal examination during routine STI check-ups in FSWs and MSM indicate that over a period of time, both health care providers and clinic attendees reached a degree of comfort and accepted it as a norm. Thus, improving health seeking behaviour and acceptable service provision is possible on a large scale.

A report of a cohort of female sex workers in Pune provided with STI services followed over a nine-year period showed declining trends of genital ulcer disease prevalence while the vaginal discharge syndrome remained stable [28]. In our analysis, all STI syndromes diagnosed amongst those who attended clinics showed a declining trend, while regular STI check-up visits increased consistently The present data show a declining trend in syndromes diagnosed possibly because of the essential service package approach followed in Avahan clinics. Though in the absence of controls this cannot be a direct measure of causality the scale of such a declining trend in STI syndromes in all six states of India could not be possible without a large intervention amongst HRGs such as Avahan.

Providing quality STI services encourage STI patients to seek care at such facilities. Quality services are technically sound and based on evidence-informed standard guidelines [29]. Though perceived quality was not measured in the present analysis, high standards were maintained by using the STI service guidelines detailed in the COGS which were developed early in the Avahan programme. Experience in STI control programmes indicates that assessment and improvement of service quality is an essential part of programme management, leading to a more effective and efficient use of resources . Recent efforts to assess the quality of STI services have relied on review of patient records, simulated patients and observational methods as data sources, which are difficult to implement in resource-constrained scenarios [30]. According to the WHO protocol for STI case management, Prevention Indicator 6 (PI 6), now renamed as (HIV-prevention indicator) PI 11 measures different components of STI case management including history taking, examination and correct treatment given as per the clinical diagnosis [31]. In the present analysis, correctness of treatment was measured by comparing the treatment packets with the syndromes diagnosed. The analysis found that correct treatment, ranging from 61% to 95%, was given for all syndromes. In comparison, another study in Nairobi, Kenya showed that correct treatment for syndrome management was given 33% of times with a range of 9% to 63% [32]. In studies carried out in the rural health districts of KwaZulu-Natal, South Africa, and correct treatment improved when treatment packs were standardized [33]. Our analysis is one of the first to report high standards of correct treatment for STIs using coded packs implemented on a large scale in India. The present analysis showed that the population coming to the clinics is changing constantly, gauging by the changing clinic attendance patterns. In addition to HRGs being socially, culturally and economically marginalized, mobility itself presents barriers to health care access [34]. Changing patterns of new individuals coming in and drop outs amongst the clinic attendees over the years reveal the dynamic nature of the cohorts being followed. This perhaps reflects mobility amongst HRG population in India. There is a gap in our understanding of the dynamics of mobility in sex work and its impact on STI prevention [35]. Hence, in India structural interventions and STI prevention strategies need to address mobility so that impact of these strategies is not mitigated.

### Limitations of the analysis

The analysis of the paper was based on clinical records and hence the findings cannot be generalized to the community at large Another key limitation of the analysis was that the individual tracking system was NGO-specific. Sex workers in India are a mobile population and often move, either for short term or longer term, from one solicitation point to another [36]. Accordingly, such individuals registered in one NGO clinic may have registered again in another NGO clinic(s) given the programme’s extensive reach in 83 of 129 districts in six states. This multiple registration may explain the over 100% clinic utilization of certain typologies of HRGs. The denominators for the size estimates were calculated by the state lead implementing partners through various research agencies, hence may not be of similar quality across the states giving rise to under or over estimates [37]. Finally, this analysis examined the data across all Avahan sites. The authors recognize that there are potential geographical variations across the state and within districts both in utilization as well as with service delivery models (e.g., static clinics, mobile clinics, and health camps) but such analyses were beyond the scope of this paper.

## Conclusions

The analysis of routine MIS data from STI clinical services shows that utilization of health services by marginalized groups can be dramatically improved with efforts to make clinics acceptable and accessible through quality services; along with outreach efforts to promote health seeking behaviour. Utilization of quality services results in improved treatment seeking behaviour and decrease in prevalence of STI syndromes.

## Competing interests

The authors declare that they have no competing interests.
